# A cross-sectional study of the relationship between community dwelling older adults’ self-perceived frailty and their electronic frailty index score

**DOI:** 10.1186/s12877-026-07386-x

**Published:** 2026-04-01

**Authors:** Victoria Barber-Fleming, Atul Anand, Heather Wilkinson, Gillian Mead

**Affiliations:** 1 Advanced Care Research Centre, College of Engineering, College of Medicine and Veterinary Medicine, University of Edinburgh, Edinburgh, UK; 2 Advanced Care Research Centre and Institute for Neuroscience and Cardiovascular Research, University of Edinburgh, Edinburgh, UK; 3 Advanced Care Research Centre, University of Edinburgh, Edinburgh, UK; 4 Usher Institute, University of Edinburgh, Edinburgh, UK

**Keywords:** Older adults, Frailty, Frailty index, Frailty screening, Perceptions, Advance care planning

## Abstract

**Background:**

Qualitative studies suggest discrepancies between older adults’ self-perceived and measured frailty. Quantification of this is limited. This study investigated the relationship between older adults’ self-perceived frailty and a measure of frailty derived from electronic heath record data (electronic Frailty Index [eFI] score). The eFI is derived routinely from available primary care electronic health record data and is based on the cumulative deficit model of frailty.

**Methods:**

One thousand people (≥ 70 years), randomly selected from a GP practice, were sent a survey, asking them to rate their frailty (ordinal and binary scale), and complete self-rated health (SRH) and PRISMA-7 questionnaires. We analysed (a) agreement between self-perceived frailty (ordinal scale) and eFI categorised frailty; (b) discrimination of self-report measures for eFI defined frailty (threshold ≥ 0.12); and (c) predictors of self-perceived frailty (logistic regressions).

**Results:**

375 people were analysed (median age 76, 51% female). Agreement was ‘fair’ between self-perceived frailty and eFI (linear weighted Kappa 0.25, quadratic weighted Kappa 0.37). Agreement was higher with linear and quadratic weighted Gwet’s second order agreement co-efficient [AC2]), (0.65 and 0.81 respectively). As eFI increased, agreement with self-perceived frailty decreased. Disagreements commonly reflected self-perceived frailty reported as less severe than eFI.

Self-perceived frailty poorly discriminated eFI defined frailty (AUC 0.59, 95%CI 0.55-0.63) as did SRH, while PRISMA-7 reached moderate discrimination (AUC 0.71, 95%CI 0.66-0.76). The optimal eFI cut-point for discriminating self-perceived frailty was 0.17.

A multivariable regression model revealed increasing age (OR 1.10 per year, 95%CI 1.02-1.18) and depression (OR 1.51, 95%CI 1.31-1.74) were associated with self-perceived frailty, however, sex, anxiety, eFI score and deprivation were not.

**Conclusions:**

The mismatch between self-perceived and eFI categorised frailty has implications for the social acceptability of screening and for meaningful engagement with frailty interventions including advance care planning.

**Supplementary Information:**

The online version contains supplementary material available at 10.1186/s12877-026-07386-x.

## Background

Health care professionals and researchers generally conceptualise frailty using either the phenotype [1] or accumulative deficit model [2]. However, qualitative studies suggests that older adults do not consider frailty in accordance with these models [3] or health care professionals [4], often meeting clinical frailty criteria but not perceiving themselves as frail [5–9].

Another way to consider how older adult’s self-perceived frailty relates to clinical assessment, is to compare frailty self-report questionnaires with measures used by health care professionals. Aiming to find less resource-intensive frailty screening, studies have compared self-rated components of the phenotype model with standard phenotype ratings [10–12]. However, self-rating *components of frailty* in this way does not capture how a person considers that frailty as a concept relates to them.

In contrast, other studies have compared self-rated frailty on an ordinal scale to measured frailty. In emergency department (ED) settings, small studies compared patient and clinician ratings on the Clinical Frailty Scale (CFS) finding moderate agreement [13, 14]. A study involving haemodialysis patients compared their self-rated frailty to phenotype frailty, finding poor agreement [15]. Asking participants to rate their own frailty in this way provides insight into how they perceive their own frailty status, however, these findings from the ED and haemodialysis clinic are unlikely to generalise to community dwelling, older adults with different health experiences. The relationship between community dwelling, older adults’ self-perceived frailty and objectively measured frailty remains an area of uncertainty.

This relationship is important to understand, as a mismatch threatens engagement of older adults in frailty interventions, including advance care planning (ACP). ACP is important for those living with frailty as they are at increased risk of sudden health deteriorations [16] with a 90% increased odds of hospitalisation (compared to non-frail) [17] and poor outcomes from unplanned admission [18]. A heightened risk of cognitive impairment [19], threatens decision-making ability for those with frailty. However, engagement in ACP may seem irrelevant to those who do not perceive themselves as frail.

It also has implications for the social acceptability of frailty screening. If older adults do not agree with their frailty screening outcome, this could challenge communication between patient and clinician, further threatening shared decision making.

We therefore aimed to enhance understanding of the relationship between older adults’ self-perceived frailty and the Electronic Frailty Index (eFI), an objective frailty screening tool commonly used in clinical practice, based on the cumulative deficit model of frailty (considering thirty-six deficits [listed in Additional file 1], which can categorise people as fit, living with mild, moderate or severe frailty, and is predictive of poor outcomes, including mortality, hospital and nursing home admission [20].

## Methods

### Study design, setting and participants

We conducted a cross-sectional survey of a random sample of 1000 community dwelling, older adults (aged ≥ 70 years) from a single GP practice in Scotland. The large, GP-run practice (10,000–12,000 patients) has an age distribution reflective of the Scottish population and a range of deprivation levels [21]. Participants were identified by the NHS Research Scotland (NRS) Primary Care Network in May 2023. Inclusion criteria included: registered with the medical practice, aged ≥ 70 years, able to engage with the documentation in English and able to give informed consent. Exclusion criteria included: significant cognitive impairment or considered to be in the last days of life. These were screened for using GP database Read-codes (reflective of a dementia diagnosis or end of life). The final list of potential participants was reviewed by a GP, who made no further exclusions. Potential participants were randomly sampled using the RAND () function in excel.

The postal survey (Additional file 2), followed by a reminder letter, asked participants to rate their own frailty by completing multiple self-report measures. A brief definition of frailty, created by the first author alongside patient and public involvement members, was provided with the survey. Surveys were returned between May-September 2023.

During 2020–2021, the GP practice undertook quality improvement project (QIP) work which identified patients with more severe frailty using clinical judgement and eFI scores and offered them GP review. Participation in this QIP work prior to the present study was recorded for data analysis.

The study was pre-registered with Open Science Framework (DOI: 10.17605/OSF.IO/5FWKG) and approved by West of Scotland Research Ethics Service (REC reference: 23/WS/0041).

### Measures and data

- Automatically generated eFI scores and their associated frailty severity group were extracted from the GP database (11 May 2023) using the data extraction tool, Scottish Primary Care Information Resource. eFI cut-points: fit = < 0.12, mild ≥ 0.12–0.24, moderate = 0.24–0.36, severe = ≥ 0.36 [22].
- Participants rated their frailty (a) on a binary scale (‘Do you think you are living with frailty? – yes/no’) and (b) on an ordinal scale (‘Please rate yourself on the following scale in relation to frailty: fit/mild/moderate/severe frailty’).
- PRISMA-7 is a seven-item, self-report questionnaire, scored by counting positive answers (marked as ‘yes’) [23]. A score of ≥ 3 indicated frailty.
- Self-Reported Health (SRH) was rated in response to the question ‘how would you rate your health status on a scale from 0 to 10?’. A score of ≤ 6 was the cut-point for frailty [24].
- The Hospital Anxiety and Depression Scale (HADS) contains two subscales - one for anxiety (HADS-A) and one for depression (HADS-D), totalling 14 questions each scored 0–3 [25]. Scores of ≥ 8 on either HADS-A or HADS-D indicated anxiety or depression respectively. Missing HADS data was imputed using the individual participants’ subscale mean value [26].

GP system data provided basic demographics including age and sex. Relative socioeconomic deprivation was obtained from postcode matching to quintiles of the Scottish Index of Multiple Deprivation (SIMD) [27].

### Study size

The sample size was agreed with the GP practice, reflecting the maximum number they felt comfortable inviting. Based on response rates from previous surveys by this practice, we anticipated a 40–50% response to single mailing, increasing after a reminder letter.

### Statistical methods

A pre-specified statistical analysis plan was followed. Participants were categorised by eFI frailty severity group. Data were summarised by median [quartile 1, quartile 3] due to distribution. Kruskal-Wallis tests were used to compare age and eFI between the groups. Fisher’s exact tests considered statistical association between group and sex, SIMD quintile, presence of an ACP and involvement in QIP work.

Basic data for participant and non-respondent groups were compared. A Brunner Munzel test compared age distributions between groups. Differences in eFI score between participants and non-responders were assessed by Mann Whitney U test and Chi-squared tests were used for comparisons of categorical data.

#### Primary analysis

Agreement between self-rated frailty (ordinal scale) and eFI categorised frailty was calculated using weighted Kappa [28]. Participants with missing data for self-rated frailty (ordinal scale) were excluded from this analysis. Due to imbalance in group sizes, a post-hoc analysis, calculated agreement using an alternative, chance-corrected agreement coefficient, AC2 [29]. A more detailed discussion of this is provided (see Additional file 3).

We conducted three sensitivity analyses, restricted to participants:

1. Without depression or anxiety on HADS.
2. Who did not have help completing the survey.
3. Not involved in prior QIP work.

#### Secondary analyses

Accuracy was assessed using the sensitivity, specificity, positive predictive value (PPV), negative predictive value (NPV), positive likelihood ratio (PLR) and negative likelihood ratio (NLR) of the following self-report measures: frailty rated on the binary scale, PRISMA-7 and SRH, using an eFI cut-point ≥ 0.12 as the reference standard. Missing self-report data resulted in exclusion from the accuracy tests relevant to that self-report measure only. Discrimination of each self-perception measure for eFI defined frailty was reported using the Area Under the Curve (AUC). The above accuracy tests were repeated considering frailty rated on the ordinal scale and with conversion to a dichotomised state (mild, moderate or severe frailty combined) in a post-hoc analysis. We additionally considered the optimal eFI cut-point at which people perceive their own frailty, using the Receiver Operating Characteristic (ROC) curve, defining this as the threshold with the maximum difference between true positive rate and false positive rate, maximising sensitivity and specificity.

Logistic regression was used to understand variables associated with self-perceived frailty. Assumptions of linearity and multi-collinearity were checked (detailed in Additional file 4). Initial univariable analyses assessed how each of the following predictors were associated with self-perceived frailty: age, sex, anxiety, depression, eFI score, SIMD quintile, presence of an ACP, involvement in QIP work. Multivariable analysis included the same predictors. Missing data resulted in exclusion from the regression calculation.

Python (v3.12.7) was used for all analyses. Statistical packages are provided in Additional file 5.

## Results

### Participant characteristics

**Fig. 1:**
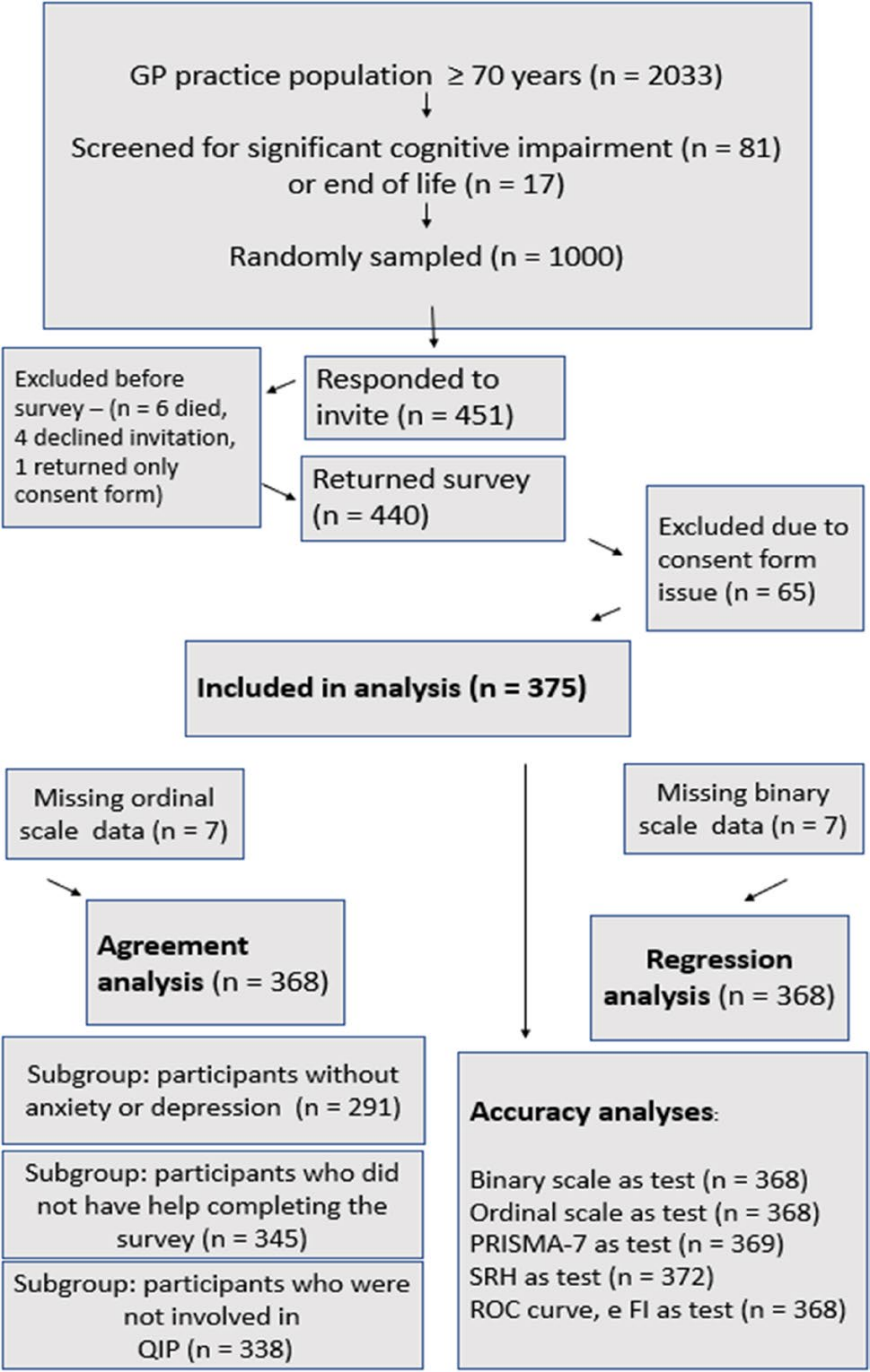
Recruitment and attrition rates with missing data numbers for each analysis. *PRISMA-7, seven-item self-report questionnaire, SRH, Self-rated health. ROC, receiver operating characteristic*

Recruitment and attrition rates are presented in Fig. 1. Of the 1000 invited participants, 451 responded and 375 could be analysed. Participants had a median age of 76, almost equal sex divide (51% female) and spread of SIMD quintiles, excluding SIMD 1 (most deprived). Participant characteristics, stratified by eFI categories, are provided in Table 1.

**Table 1:** Characteristics of participants in each of the eFI categorised frailty groups

	Overall (*n* = 375)	Fit (*n* = 177)	Mild frailty (*n* = 138)	Moderate frailty (*n* = 47)	Severe frailty (*n* = 13)	*p* value
Age (years)						< 0.001
Median	76.0	74.0	77.0	77.0	87.0	
(Quartile 1, Quartile 3)	(72.0, 80.0)	(72.0, 77.0)	(74.0, 81.8)	(74.0, 84.5)	(78.0, 89.0)	
Sex						0.17
Female	193 (51)	95 (54)	62 (45)	27 (57)	9 (69)	
Male	182 (49)	82 (46)	76 (55)	20 (43)	4 (31)	
SIMD Quintile						0.10
SIMD 1 (most deprived)	0	0	0	0	0	
SIMD 2	73 (19)	32 (18)	21(15)	16 (34)	4 (31)	
SIMD 3	40 (11)	23 (13)	13 (9)	2 (4)	2 (15)	
SIMD 4	111 (30)	53 (30)	43 (31)	14 (30)	1 (8)	
SIMD 5 (least deprived)	151 (40)	69 (39)	61 (44)	15 (32)	6 (46)	
eFI						< 0.001
Median	0.14	0.08	0.17	0.28	0.39	
(Quartile 1, Quartile 3)	(0.08, 0.19)	(0.03, 0.11)	(0.14, 0.19)	(0.25, 0.31)	(0.36, 0.42)	
ACP present	53 (14)	10 (6)	14 (10)	19 (40)	10 (77)	< 0.001
Involved in QIP	30 (8)	0	6 (4)	16 (34)	8 (62)	< 0.001

Figures are number and % unless otherwise defined. Abbreviations: *SIMD *Scottish Index of Multiple Deprivation [27], *ACP *advance care plan, *QIP *quality improvement project.

The median eFI score was 0.14, consistent with mild frailty. Frailer groups were older: median age 87 [78, 89] years in severely frail vs. 74 [72, 77] years in fit patients, *p* < 0.001. No differences were observed in sex or SIMD quintile. Frailer groups had a higher proportion of people with an ACP in place (77% of those categorised ‘severe frailty vs. 6% categorised as ‘fit’, p = < 0.001) and a higher proportion who had been involved in the QIP (62% of those categorised ‘severe frailty’ vs. 0% categorised ‘fit’, p = < 0.001).

The participant versus non respondent comparisons (Additional file 6) revealed that the participants were marginally younger (median age 76 [72, 80] years vs. 76 [73, 83] years in non-respondents [*p* = 0.01]), and less deprived (< 0.001) than the non-respondents. However, there was no difference in distribution of eFI between groups and no association between sex and group. 

### Agreement

**Fig. 2:**
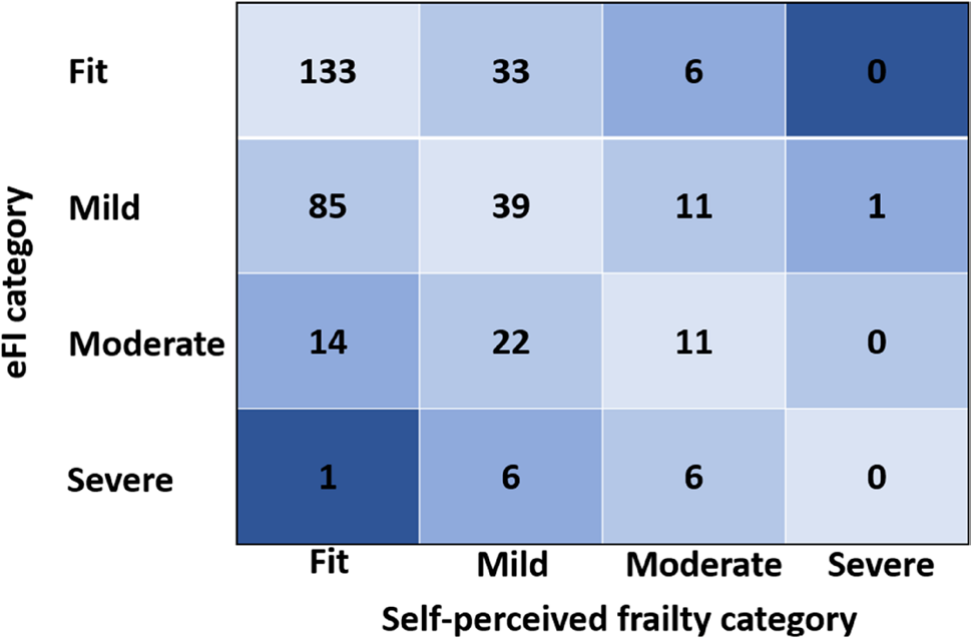
Confusion matrix for the overall group

Half of the participants self-rated their frailty level in agreement with their eFI severity. Figure 2 is the confusion matrix for the overall group. The numbers in each box represent the number of participants categorised by the confusion matrix. The diagonal line shaded lightest blue represents perfect agreement between eFI and self-perceived frailty. Darker shades of blue represent larger disagreements. Figure 2 demonstrates that most of the agreements were between people categorised by eFI as ‘fit’ who also perceived themselves as ‘fit’. As eFI categorised frailty severity increased, agreement with self-perceived frailty decreased: 77% of those categorised as fit; 29% of mildly frail; 23% of moderately frail and 0% of those categorised as severely frail. More participants underrated their frailty compared to eFI (36%) than overrated it (14%).

Linear weighted kappa (k = 0.25) and quadratic weighted kappa (k = 0.37) both revealed fair agreement between self-rated and eFI categorised frailty (Table 2). AC2 resulted in higher agreement (linear weighted AC2 = 0.65, quadratic weighted AC2 = 0.81), reflecting differences in handling of chance agreement by the coefficients. This is discussed further in Additional file 3.

**Table 2:** Primary outcome data: agreement between eFI and perceived frailty with sensitivity analyses. Kappa and AC2 co-efficients with 95% confidence intervals and p values

	*n*	% Agreement	Linear weighted Kappa	Quadratic weighted Kappa	Linear weighted AC2	Quadratic weighted AC2
Overall	368	49.7%	0.25 (0.17–0.32) *p* < 0.001	0.37 (0.28–0.46) *p* < 0.001	0.65 (0.60–0.69) *p* < 0.001	0.81 (0.78–0.85) *p* < 0.001
Participants without anxiety or depression	291	53.3%	0.21 (0.12–0.29) *p* < 0.001	0.31 (0.20–0.42) *p* < 0.001	0.70 (0.65–0.75) *p* < 0.001	0.85 (0.82–0.88) *p* < 0.001
Participants who did not have help completing the survey	345	50.4%	0.23 (0.15–0.31) *p* < 0.001	0.34 (0.25–0.43) *p* < 0.001	0.66 (0.61–0.71) *p* < 0.001	0.82 0.79–0.86) *p* < 0.001
Participants who were not involved in QIP	338	52.1%	0.20 (0.12–0.29) *p* < 0.001	0.29 (0.19–0.39) *p* < 0.001	0.69 (0.64–0.73) *p* < 0.001	0.84 (0.81–0.87) *p* < 0.001

*AC2* Gwet’s second order agreement co-efficient, *QIP *quality improvement project

Sensitivity analyses did not demonstrate major changes in patterns of agreement after removing those with anxiety or depression, those who had help completing the survey, or those who were involved in QIP work.

### Accuracy

Self-perceived frailty (rated on binary scale) had low sensitivity (0.27, 95%CI 0.23–0.32) and high specificity (0.91, 95%CI 0.88–0.94) for eFI defined frailty (Table 3), with poor overall discrimination (AUC 0.59, 95%CI 0.55–0.63). Similarly low sensitivity and high specificity were observed with self-rated health. Self-perceived frailty rated on an ordinal scale, then converted to binary, had a higher sensitivity (0.49, 95%CI 0.44–0.54), lower specificity (0.77, 95%CI 0.73–0.82), and higher AUC (0.64, 95%CI 0.59–0.69) than the binary scale. Of the 299 people who rated themselves as ‘not living with frailty’ on the binary scale, 19% also rated themselves as ‘mildly frail’ on the ordinal scale.

**Table 3:** Accuracy Tests when eFI measured frailty (cut-point of ≥ 0.12) is the reference standard

Index Test	Cut-point	Sensitivity	Specificity	PPV	NPV	PLR	NLR
Binary scale (Self -perception)	Binary	0.27 (0.23-0.32)	0.91 (0.88-0.94)	0.77 (0.72–0.81)	0.53 (0.47–0.58)	2.94 (1.61–5.37)	0.80 (0.44–1.47)
Ordinal scale converted to binary (Self-perception)	Mild/ Moderate/ Severe	0.49 (0.44-0.54)	0.77 (0.73–0.82)	0.71 (0.66–0.76)	0.57 (0.52–0.62)	2.16 (1.37–3.40)	0.66 (0.42–1.04)
PRISMA-7	≥ 3	0.49 (0.44-0.55)	0.86 (0.82–0.89)	0.79 (0.75–0.83)	0.60 (0.55–0.65)	3.46 (2.08–5.76)	0.59 (0.35–0.98)
SRH	≤ 6	0.23 (0.19 –0.27)	0.96 (0.94–0.98)	0.87 (0.83–0.90)	0.53 (0.48–0.58)	5.84 (2.55–13.33)	0.80 (0.35–1.83)

95% confidence intervals provided. Prevalence of eFI categorised frailty was 52-53%. Abbreviations: *PPV *positive predictive value, *NPV *negative predictive value, *PLR *positive likelihood ratio, *NLR *negative likelihood ratio

PRISMA-7 had a similar sensitivity to binary conversion of ordinal self-perceived frailty (0.49, 95%CI 0.44–0.55), but better specificity (0.86, 95%CI 0.82–0.89) with associated better discrimination (AUC 0.71, 95%CI 0.66–0.76). The ROC curves are presented in Additional file 7.

The optimal eFI cut-point for discriminating self-perceived frailty was 0.17 (see Additional file 8). Applying this threshold to the study participants, 157 (42%) were classified as frail, compared to 198 (53%) participants using a standard eFI threshold of 0.12 to define frailty.

### Regression

Univariable analyses demonstrated that age, anxiety, depression, eFI category, presence of an ACP and involvement in the QIP work were all predictive of self-perceived frailty.

Age and depression remained significant in the multivariable analysis. For every additional year of age, participants had 10% increased odds of self-perceived frailty (OR 1.10, 95%CI 1.02–1.18). For every point increase on the HADS depression scale, the participants had 50% increased odds of self-perceived frailty (OR 1.51, 95%CI 1.31–1.74).

There was a trend towards increased self-perceived frailty with increased eFI categorised frailty. Using eFI defined ‘fit’ participants as a reference group, the odds of self-perceived frailty increased from mild frailty (OR 1.16, 95%CI 0.49–2.74), to moderate frailty (OR 2.13, 95%CI 0.73–6.19) and severe frailty (OR 2.71, 95%CI 0.47–15.76).

Anxiety, presence of an ACP, or having been involved in the QIP work did not retain significance in the full model. Full results of the regression analyses are displayed in Additional file 9.

## Discussion

We analysed the relationship between self-perceived frailty and a frailty screening measure for a large group of community dwelling, older adults. We found a mismatch between the two, which has important implications for the design of frailty-attuned services. In general, older adults were more optimistic about their frailty than the eFI. Half of the participants rated their frailty differently to their eFI status, with a minority of people at every level of eFI-defined frailty agreeing with this assessment. Increasing age and markers of depression increased the likelihood of self-perceiving frailty. Taken together, our findings suggest that interventions that are targeted towards ‘frail’ older adults risk limited engagement.

Studies comparing self-rated frailty with measured frailty in other contexts, have also found imperfect agreement. Agreement was slight in one small study of 146 haemodialysis patients [15]. In ED studies, older adults and health care professionals’ ratings on the CFS reached moderate agreement [13, 14]. Although these results do not generalise to community dwelling older adults, due to different health experiences which likely influence perceptions of frailty, they are consistent with ours.

Nearly 1 in 5 of those who rated themselves as ‘not living with frailty’ also rated themselves as ‘mildly frail’ when offered more options. The additional granularity from considering frailty on an ordinal rather than binary scale resulted in better ability to distinguish eFI defined frailty. A binary option appears too simplistic for frailty, a complex concept viewed differently by individual older people [6, 8, 30]. Those aiming to maximise recruitment of people with frailty using self-report measures might consider wider ranging scales.

SRH and PRISMA-7 have previously been recommended for frailty assessment in the community [24]. However, SRH performed poorest here, with low sensitivity (23%) for discriminating eFI categorised frailty. This contrasts to 83% sensitivity in studies of SRH in relation to phenotypic frailty [31] and evidence that poor SRH predicts future phenotype model frailty [32, 33]. Differences in definitions and measures of frailty are likely to impact agreement with self-perceived frailty. Individuals with physical limitations related to frailty, as expressed in the phenotypic model, are perhaps more likely to report poor health. We found a disconnect between SRH and eFI, perhaps reflecting that early frailty status on eFI may represent multimorbidity deficits without necessarily having profound impact on physical function. PRIMSA-7 was the best performing self-report measure for distinguishing eFI defined frailty. It has been shown elsewhere to have higher sensitivity and similar specificity (both 83%) for identifying phenotypic frailty [31].

Recently a systematic review found the eFI was the best performing electronic frailty measure compared to phenotype frailty, however, its high false positive rate would screen an overwhelming number of people as frail [34]. Maximised performance was achieved with a higher eFI threshold of 0.21 to determine frailty [35]. Our results suggest an optimised eFI threshold of 0.17 for self-perceived frailty. Both are significantly higher than the 0.12 threshold for frailty adopted in clinical practice [20]. Applying the higher cut-point based on perceived frailty to our sample, reduced the proportion identified as frail by over 10%. This could be more feasible for resource allocation and would target people more in line with how they perceive their own frailty.

Importantly, we found an association between measures of depression and self-perceived frailty. This aligns with older adults’ views that there is a psychological element to frailty [36] and that negative attitude and mood can have a detrimental effect on frailty [6]. Further, older adults can base their self-perceptions of frailty on how they feel emotionally [36], justifying a lack of frailty due to their ‘positive spirit’ [5]. A bidirectional, causal association between frailty and depression has been suggested, however, the underlying mechanisms remain unclear [37]. Self-perceptions may be important here, as self-perceived ageing has been shown to predict frailty directly and indirectly by influencing depression [38] and people living with frailty, who perceive themselves as frail, describe more feelings of depression than those who consider themselves not to be frail [7].

### Strengths

Our sample size of 375 older adults is larger than many similar studies [13–15, 39] and represents of a community-dwelling general population rather than restricted sampling, for example in ED studies.

### Limitations

The survey response rate (45.1%), is comparative to other postal questionnaires that have asked older adults to self-rate components of phenotype frailty, which achieved 36.6% [40] and 55% response rates [41]. The impact of non-responders was considered, and those who took part were marginally younger and less deprived than non-respondents. However, there was no difference in distribution of frailty severity or sex between groups. The GP practice did not represent the most socioeconomically deprived quintile, limiting generalisability to this group.

Although the proportion of dementia that is diagnosed is increasing, estimated in England to be over 80% [42], this still leaves a large number of community dwelling older adults who are living with undiagnosed dementia. It is therefore likely, that despite screening the list of potential participants for cognitive impairment using GP database read codes reflective of dementia, alongside a GP screening this list, participants with cognitive impairment could have been included in the analysis. Potentially, cognitive impairment could impact on self-perceived frailty. However, the planned exclusion of those with cognitive impairment is also a limitation. Future research should aim to include those with cognitive impairment, including a measure of cognitive function in order to consider the impact on self-perceived frailty, however, this was out with the scope of the current study.

We also acknowledge that the eFI is not a gold standard test for frailty, but rather a population level risk stratification tool. Ultimately, frailty represents diminishing reserves that worsen outcomes in response to acute stress. We do not have outcomes data for our cohort to determine whether self-perceived frailty or eFI is better at predicting important frailty endpoints.

### Implications for clinical practice and research

For those with severe frailty, certain treatments are burdensome without benefit, and patient wishes are paramount in decision making [19]. None of the participants categorised (by eFI) as severely frail rated themselves in this way. This mismatch threatens engagement with, and informed decision making within, the ACP process. Clinicians should approach ACP with patients identified as frail, with an awareness that they may not perceive themselves as such.

Population screening for frailty risks public unacceptability partly in relation to qualitative evidence that older adults do not identify as frail [43]. Our findings of a discrepancy between screening outcomes and self-perceived frailty support and quantify this concern.

A patient-centred approach incorporating self-perceived frailty into clinical screening merits further research. To understand the predictive value of self-perceived frailty, in line with research which shows that SRH predicts mortality [44], future research should consider the relationship between self-perceived frailty and patient centred outcomes. A second version of the eFI has recently been developed [45]. It is important to understand whether this has improved agreement with self-perceived frailty in future research.

## Conclusion

Older adults did not perceive their own frailty in line with an objective measure of their frailty. The mismatch was most pronounced for those with severe frailty. Older adults had a higher threshold than the eFI for perceiving their frailty. This has important implications for their meaningful engagement in frailty interventions, including ACP. Incorporating this understanding into screening may save resources and be more acceptable to older people.

## Supplementary Information

Supplementary Material 1: Additional file 1. List of deficits included in the Electronic Frailty Index. Additional file 3. Considering the impact of the unbalanced sample on agreement calculations. Additional file 4. Details of assumption checking. Additional file 5. Packages and functions for statistical analysis. Additional file 6. Characteristics of included participants versus non-respondents. Additional file 7. AUC derived using eFI categorised frailty as the state variable and self-rating measures as test variables. Additional file 8. ROC curve for Self-perceived frailty (binary scale) as ‘state’ variable and eFI (continuous scale) as ‘test’ variable. Additional file 9. Results of univariable and multivariable logistic regression

Supplementary Material 2: Additional file 2. Survey.

## Data Availability

The datasets generated and analysed during the current study are available in Edinburgh DataShare repository at 10.7488/ds/8100.

## Abbreviations

eFI: Electronic frailty index
SRH: Self-reported health
AC2: Gwet’s second order agreement co-efficient
ED: Emergency department
CFS: Clinical frailty scale
ACP: Advance care planning
QIP: Quality improvement project
HADS: Hospital anxiety and depression scale
SIMD: Scottish index of multiple deprivation
PPV: Positive predictive value
NPV: Negative predictive value
PLR: Positive likelihood ratio
NLR: Negative likelihood ratio
AUC: Area under the curve
ROC: Receiver Operating Characteristic
