# Supplementary material for: A cross-sectional study of the relationship between community dwelling older adults’ self-perceived frailty and their electronic frailty index score
Source: BMC Geriatr. 2026 Apr 1;26:683. doi: 10.1186/s12877-026-07386-x (PMC13185184; doi:10.1186/s12877-026-07386-x)
Supplement: Supplementary file 1 — Supplementary Material 1: Additional file 1. List of deficits included in the Electronic Frailty Index. Additional file 3. Considering the impact of the unbalanced sample on agreement calculations. Additional file 4. Details of assumption checking. Additional file 5. Packages and functions for statistical analysis. Additional file 6. Characteristics of included participants versus non-respondents. Additional file 7. AUC derived using eFI categorised frailty as the state variable and self-rating measures as test variables. Additional file 8. ROC curve for Self-perceived frailty (binary scale) as ‘state’ variable and eFI (continuous scale) as ‘test’ variable. Additional file 9. Results of univariable and multivariable logistic regression [file 12877_2026_7386_MOESM1_ESM.docx]

**Additional files 1 and 3-9**

**Additional file 1.** List of deficits included in the Electronic Frailty Index [1]

Activity limitation

Anaemia & haematinic deficiency

Arthritis

Atrial fibrillation

Cerebrovascular disease

Chronic kidney disease

Diabetes

Dizziness

Dyspnoea

Falls

Foot problems

Fragility fracture

Hearing impairment

Heart failure

Heart valve disease

Housebound

Hypertension

Hypotension/syncope

Ischaemic heart disease

Memory & cognitive problems

Mobility & transfer problems

Osteoporosis

Parkinsonism & tremor

Peptic ulcer

Peripheral vascular disease

Polypharmacy

Requirement for care

Respiratory disease

Skin ulcer

Sleep disturbance

Social vulnerability

Thyroid disease

Urinary incontinence

Urinary system disease

Visual impairment

Weight loss & anorexia

**Reference**

1. Clegg A, Bates C, Young J, Ryan R, Nichols L, Ann Teale E, et al. Development and validation of an electronic frailty index using routine primary care electronic health record data. Age and Ageing. 2016; 45(3):353-60. <https://doi.org/10.1093/ageing/afw039>

**Additional file 3.** Considering the impact of the unbalanced sample on agreement calculations

The UK population, when split into eFI categories, results in categories which are of different sizes, with a larger fit group and smaller severely frail group [1]. In keeping with population norms, our sample had a similar distribution (Figure 1). A limitation of Kappa is that such unbalanced category proportions impact on its calculation for chance agreement, which is calculated from the marginal probabilities table [2].

Supplementary Fig 1. Overall group split into eFI categories (an ‘unbalanced sample’)

###
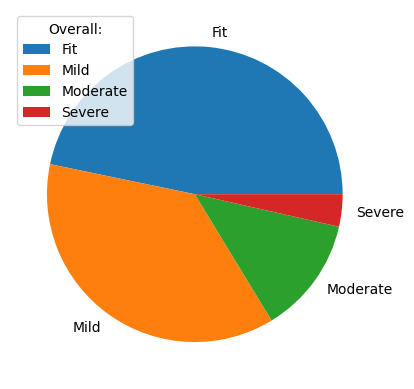


### As such, a post-hoc analysis, calculated agreement using an alternative, chance-corrected agreement coefficient, AC2 [3]. AC2 resulted in greater agreement than Kappa. This reflects the fact that Kappa and AC2 handle the calculation for chance agreement differently and are influenced by the unbalanced sample in different ways. Weights applied to both Kappa and AC2: Linear weights = 0, 0.33, 0.67, 1. Quadratic weights = 0, 0.56, 0.89. 1.

**References**

1. Morales DR, Guthrie B, Downes TJ, McAllister DA, Hanlon P. Applicability of the electronic frailty index in younger and older adults in England: a population-based cohort study. Lancet Healthy Longev. 2025;6(8):100752. <https://doi.org/10.1016/j.lanhl.2025.100752>.

2. Feinstein AR, Cicchetti DV. High agreement but low Kappa: I. the problems of two paradoxes. Journal of Clinical Epidemiology. 1990 1990/01/01/;43(6):543-9. <https://doi.org/10.1016/0895-4356(90)90158-l>.

3. Gwet KL. Handbook of Inter-Rater Reliability: The Definitive Guide to Measuring the Extent of Agreement among Raters. Fourth ed. United States of America: AgreeStat Analytics, 2014.

**Additional file 4.** Details of assumption checking

The assumption of linearity was checked both using Box-Tidwell and visual plotting. Apply the Box-Tidwell test found that the predictor variable representing ‘depression’ violated the assumption of linearity. However, on plotting the log odds of the predicted probability of self-perceived frailty against depression score, there appeared to be a linear relationship with a restricted range (due to participants not scoring the highest possible depression scores). As depression was significant and influential on univariable analyses, it was included in the overall model.

**Additional file 5.** Packages and functions for statistical analysis

The main packages and functions used: Pandas v2.2.2, Numpy v1.26.4, Statsmodels v0.14.2, sklearn v1.5.1, scipy v1.12.0 and v1.16.0, irrCAC package.

Demographic calculations**:** SciPy v1.12.0: stats.shapiro ,stats.kruskal, fisher_exact (SciPy v1.16.0), stats.levene, stats.mannwhitneyu, stats.brunnermunzel, chi2_contingency.

Agreement calculations**:** irrCAC package: Gwet, Fergadis. irrCAC. 2021. [https://irrcac.readthedocs.io/en/latest/usage/usage_table_data.html](https://irrcac.readthedocs.io/en/latest/usage/usage_table_data.html%20) (17 July 2025, last accessed).

Accuracy calculations: sklearn.metrics.confusion_matrix, sklearn.metrics.roc_curve, metrics.auc, confidenceinterval: roc_auc_score (Gildenblat, J. A python library for confidence intervals. 2023. <https://github.com/jacobgil/confidenceinterval>, (17 July 2025, last accessed). Code for calculating optimal eFI cut point for self-perceived frailty adapted from: Stack overflow (<https://stackoverflow.com/questions/28719067/roc-curve-and-cut-off-point-python> (17 July 2025, last accessed).

Regression calculations: statsmodels.formula.api: smf.logit model, statsmodels.stats.outliers_influence: variance_inflation_factor. Code for odds ratios and confidence intervals adapted from: Villazon, A. Logistic regression in Python with Statsmodels. 2021. <https://www.andrewvillazon.com/logistic-regression-python-statsmodels/> (17 July 2025, last accessed).

**Additional file 6.**

Supplementary Table 1. Characteristics of included participants versus non-respondents.

|  | **Included participants**  **(n = 375)** | **Non-respondents**  **(n = 549)** | p value |
| --- | --- | --- | --- |
| **Age (years)**  Median  (Quartile 1, Quartile 3) | 76.00  (72.00, 80.00) | 76.00  (73.00, 83.00) | 0.01 |
| **Sex**  Female  Male | 193 (51%)  182 (49%) | 310 (56%)  239 (44%) | 0.13 |
| **SIMD Quintile**  SIMD 1  (Most deprived)  SIMD 2  SIMD 3  SIMD 4  SIMD 5  (Least deprived) | 0  73 (19%)  40 (11%)  111 (30%)  151 (40%) | 0  185 (34%)  65 (12%)  130 (24%)  164 (30%)  Missing data = 5 (1%) | <0.001 |
| **eFI**  Median  (Quartile 1, Quartile 3) | 0.14  (0.08, 0.19) | 0.14  (0.08, 0.22) | 0.07 |

Figures are number and % unless otherwise defined.

**Additional file 7**.

Supplementary Table 2. AUC derived using eFI categorised frailty as state variable and self-rating measures as test variables.

|  | AUC | ROC |
| --- | --- | --- |
| **Binary scale** | 0.59  (0.55-0.63) | 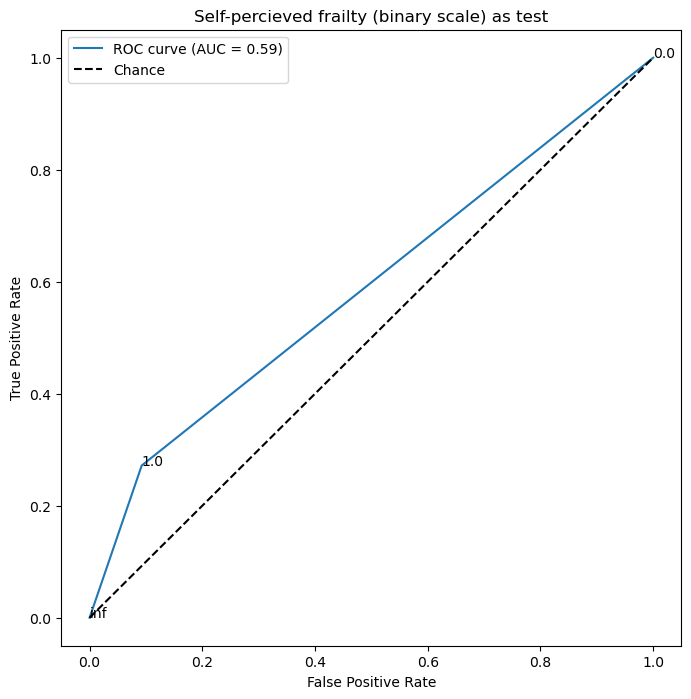 |
| **Ordinal scale** | 0.64  (0.59-0.69) | 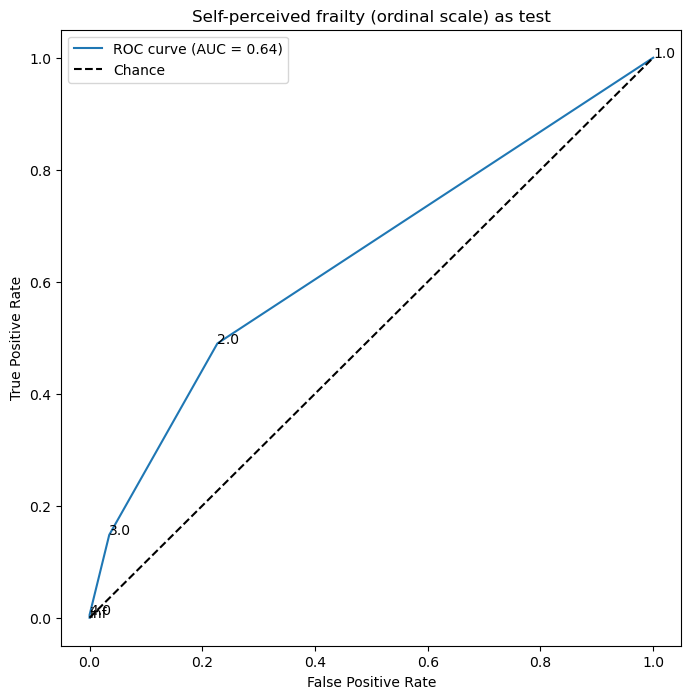 |
| **PRISMA-7** | 0.71  (0.66-0.76) | 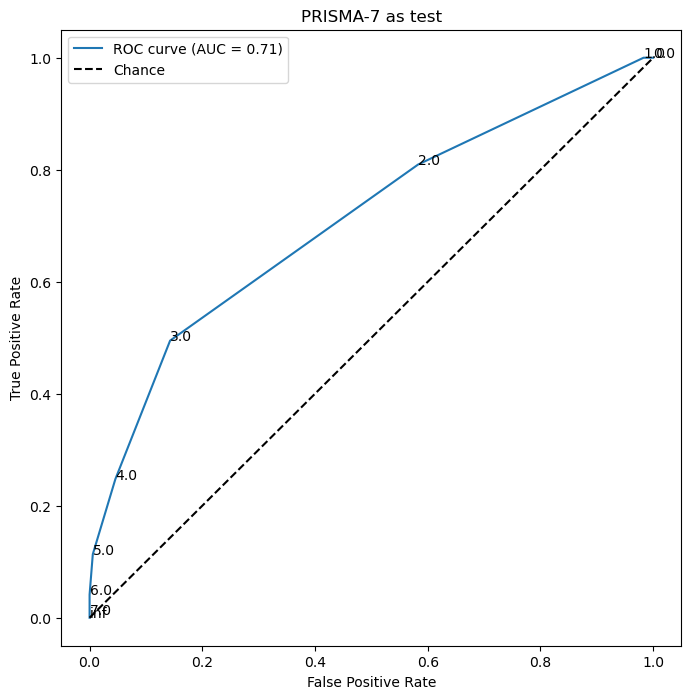 |
| **SRH** | 0.30  (0.24-0.35) | 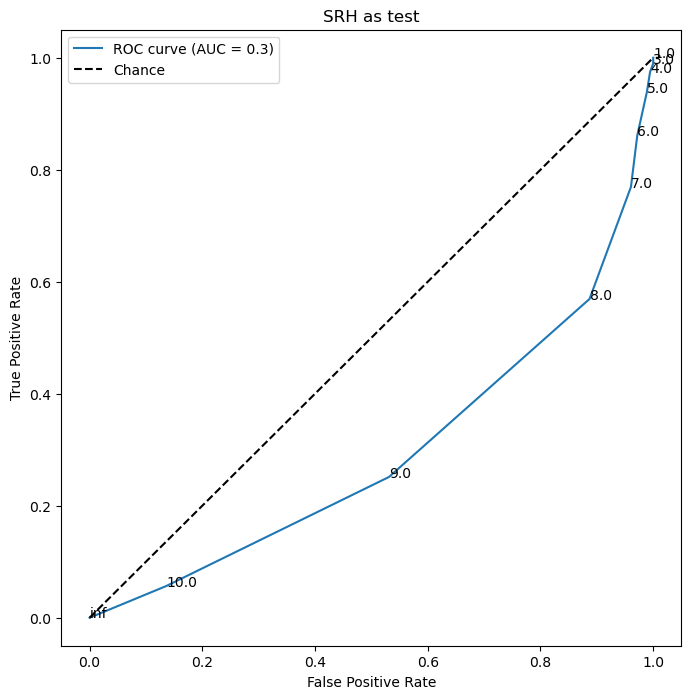 |

eFI categorised frailty as the state variable (binary cut point of ≥ 0.12)

**Additional file 8**.

Supplementary Fig 2. ROC curve for Self-perceived frailty (binary scale) as ‘state’ variable and eFI (continuous scale) as ‘test’ variable.


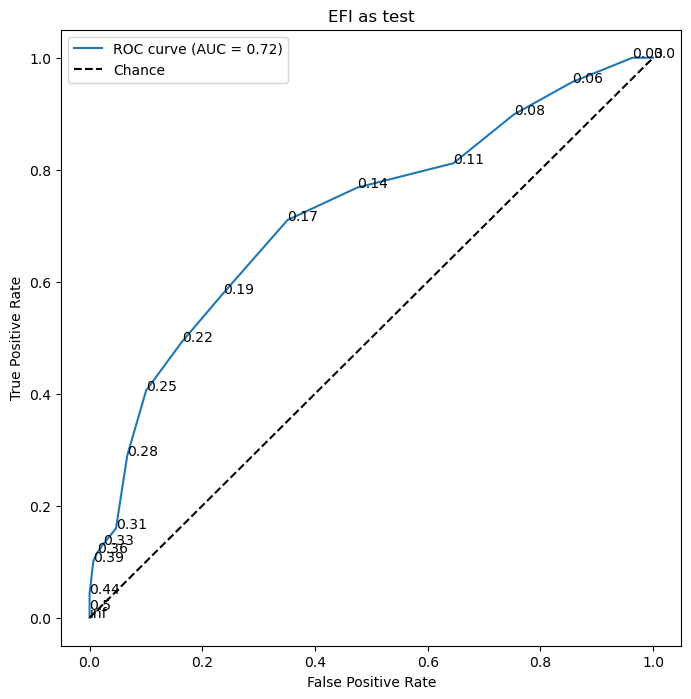


Optimal eFI cut point for self-perceived frailty = 0.17.

**Additional file 9.** Supplementary Table 3. Results of univariable and multivariable logistic regressions. Odds ratios are presented with 95% confidence intervals and p values. Multivariable model goodness of fit: Pseudo-R-squared = 0.37, p value = <0.001.

|  | **Univariable regressions** | **Multivariable regression** |
| --- | --- | --- |
| **Age** | 1.12 (1.07-1.17)  P<0.001 | 1.10 (1.02-1.18)  p = 0.01 |
| **Sex** | 0.91 (0.54-1.53)  p = 0.71 | 0.81 (0.39 -1.67)  P = 0.57 |
| **Anxiety** | 1.27 (1.18 -1.37)  p<0.001 | 1.08 (0.96-1.21)  p = 0.20 |
| **Depression** | 1.59 (1.43 -1.78)  p<0.001 | 1.51 (1.31-1.74)  p<0.001 |
| **eFI categorised frailty**  (Fit = reference)  Mild | 2.19 (1.12-4.29)  p = 0.02 | 1.16 (0.49-2.74)  p = 0.73 |
| Moderate | 7.55 (3.47-16.42)  p<0.001 | 2.13 (0.73-6.19)  p = 0.16 |
| Severe | 19.62 (5.32-72.43)  p<0.001 | 2.71 (0.47 -15.76)  p = 0.27 |
| **SIMD**  (No participants in SIMD 1)  (SIMD 2 = reference)  SIMD 3 | 0.75 (0.29-1.92)  p = 0.55 | 1.12 (0.32-3.91)  p = 0.86 |
| SIMD 4 | 0.74 (0.36-1.52)  p = 0.41 | 0.97 (0.37-2.57)  p = 0.95 |
| SIMD 5 (least deprived) | 0.52 (0.26-1.04)  p = 0.06 | 0.88 (0.34-2.27)  p = 0.79 |
| **Presence of an ACP** | 4.84 (2.57-9.12)  p<0.001 | 2.55 (0.91-7.16)  p = 0.08 |
| **Involved in QIP work** | 6.11 (2.75-13.57)  p<0.001 | 0.95 (0.24 -3.87)  p = 0.95 |

*Abbreviations: SIMD, Scottish Index of Multiple Deprivation, ACP, advance care plan, QIP, quality improvement project.*
