# Supplementary material for: A cross-sectional study of the relationship between community dwelling older adults’ self-perceived frailty and their electronic frailty index score
Source: BMC Geriatr. 2026 Apr 1;26:683. doi: 10.1186/s12877-026-07386-x (PMC13185184; doi:10.1186/s12877-026-07386-x)
Supplement: Supplementary file 2 — Supplementary Material 2: Additional file 2. Survey. [file 12877_2026_7386_MOESM2_ESM.docx]

**Additional file 2.** Survey

**Frailty is when a person has less resilience to becoming unwell and is more vulnerable to medical illness. Although more common as we get older, frailty it is not inevitably part of ageing and some older adults are fit while others live with frailty.**

**Please answer the following questions.**

1. **Do you think you are living with frailty?**

Yes |  No

1. **Please rate yourself on the following scale in relation to frailty:**

Fit

Mildly frail

Moderately frail

Severely frail

1. **How would you rate your health status, from 0-10?** (Note 10 represents best possible health).

0  1  2  3  4  5  6  7  8  9  10

**PRISMA-7 Questions (Please answer Yes or No)**

1. **Are you more than 85 years old?**

Yes |  No

1. **Are you male?**

Yes |  No

1. **In general, do you have any health problems that require you to limit your activities?**

Yes |  No

1. **Do you need someone to help you on a regular basis?**

Yes |  No

1. **In general, do you have any health problems that require you to stay at home?**

Yes |  No

1. **In case of need, can you count on someone close to you?**

Yes |  No

1. **Do you regularly use a cane, a walker or a wheelchair to move about?**

Yes |  No

**Hospital Anxiety and Depression Scale Questions**

**Please read each item and select the reply which comes closest to how you have been feeling in the past week. Don't take too long over your replies: your immediate reaction to each item will probably be more accurate than a long thought out response. The researchers will not inform your GP of these scores but please do speak to your GP if you are concerned about your mood.**

1. **I feel tense or 'wound up':**

Most of the time

A lot of the time

From time to time, occasionally

Not at all

1. **I still enjoy the things I used to enjoy:**

Definitely as much

Not quite so much

Only a little

Hardly at all

1. **I get a sort of frightened feeling as if something awful is about to happen:**

Very definitely and quite badly

Yes, but not too badly

A little, but it doesn't worry me

Not at all

1. **I can laugh and see the funny side of things:**

As much as I always could

Not quite so much now

Definitely not so much now

Not at all

1. **Worrying thoughts go through my mind:**

A great deal of the time

A lot of the time

From time to time but not too often

Only occasionally

1. **I feel cheerful:**

Not at all

Not often

Sometimes

Most of the time

1. **I can sit at ease and feel relaxed:**

Definitely

Usually

Not often

Not at all

1. **I feel as if I am slowed down:**

Nearly all the time

Very often

Sometimes

Not at all

1. **I get a sort of frightened feeling like 'butterflies' in the stomach:**

Not at all

Occasionally

Quite often

Very often

1. **I have lost interest in my appearance:**

Definitely

I don't take so much care as I should

I may not take quite as much care

I take just as much care as ever

1. **I feel restless as if I have to be on the move:**

Very much indeed

Quite a lot

Not very much

Not at all

1. **I look forward with enjoyment to things:**

As much as ever I did

Rather less than I used to

Definitely less than I used to

Hardly at all

1. **I get sudden feelings of panic:**

Very often indeed

Quite often

Not very often

Not at all

1. **I can enjoy a good book or radio or TV programme:**

Often

Sometimes

Not often

Very seldom

**Please indicate if you had help to complete this survey**

Yes |  No
